# Cerebral misery perfusion diagnosed using hypercapnic blood-oxygenation-level-dependent contrast functional magnetic resonance imaging: a case report

**DOI:** 10.1186/1752-1947-4-54

**Published:** 2010-02-18

**Authors:** Adam L Gordon, Stephen Goode, Olympio D'Souza, Dorothee P Auer, Sunil K Munshi

**Affiliations:** 1 Department of Stroke Medicine, Nottingham University Hospitals (City Campus), Hucknall Road, Nottingham, UK; 2 Division of Rehabilitation and Ageing, University of Nottingham, Room B98, Medical School, Queens Medical Centre, Nottingham, UK; 3 Division of Neuroradiology, University of Nottingham, Medical School, Queens Medical Centre, Nottingham, UK; 4 Division of Academic Radiology, University of Nottingham, Medical School, Queens Medical Centre, Nottingham, UK

## Abstract

**Introduction:**

Cerebral misery perfusion represents a failure of cerebral autoregulation. It is an important differential diagnosis in post-stroke patients presenting with collapses in the presence of haemodynamically significant cerebrovascular stenosis. This is particularly the case when cortical or internal watershed infarcts are present. When this condition occurs, further investigation should be done immediately.

**Case presentation:**

A 50-year-old Caucasian man presented with a stroke secondary to complete occlusion of his left internal carotid artery. He went on to suffer recurrent seizures. Neuroimaging demonstrated numerous new watershed-territory cerebral infarcts. No source of arterial thromboembolism was demonstrable. Hypercapnic blood-oxygenation-level-dependent-contrast functional magnetic resonance imaging was used to measure his cerebrovascular reserve capacity. The findings were suggestive of cerebral misery perfusion.

**Conclusions:**

Blood-oxygenation-level-dependent-contrast functional magnetic resonance imaging allows the inference of cerebral misery perfusion. This procedure is cheaper and more readily available than positron emission tomography imaging, which is the current gold standard diagnostic test. The most evaluated treatment for cerebral misery perfusion is extracranial-intracranial bypass. Although previous trials of this have been unfavourable, the results of new studies involving extracranial-intracranial bypass in high-risk patients identified during cerebral perfusion imaging are awaited.

Cerebral misery perfusion is an important and under-recognized condition in which emerging imaging and treatment modalities present the possibility of practical and evidence-based management in the near future. Physicians should thus be aware of this disorder and of recent developments in diagnostic tests that allow its detection.

## Introduction

Cerebral misery perfusion (CMP) was first described by Baron in 1981 [1] and represents a failure of cerebral autoregulation. CMP has been associated with decreased cerebral perfusion pressures in extracranial and intracranial atheromatous diseases, complete carotid artery occlusion, and Moyamoya disease [2]. Baron's initial description was of transient limb weakness and collapse associated with changes in patients' posture [1]. Subsequent case reports have replicated this description.

Positron emission tomography (PET) scanning has allowed us to follow the progression of cerebral haemodynamic impairment in misery perfusion through the measurement of regional cerebral blood flow (rCBF) and tissue oxygen extraction fraction (OEF) [3]. The hypothesized pathophysiology is outlined in Table 1.

**Table 1:** Pathophysiology of Cerebral Misery Perfusion (Stage 1 to 3 Cerebral Haemodynamic Impairment)

Stage 1 (Cerebrovascular autoregulation)	Any fall in regional cerebral perfusion pressure (rCPP) is matched by a fall in regional cerebrovascular resistance (rCBR) in order to maintain regional cerebral blood flow (rCBF). This is accommodated by vasodilatation and an attendant increase in regional cerebral blood volume (rCBV). Oxygen extraction factor (OEF) remains constant.
Stage 2 (Misery Perfusion)	The capacity for compensatory vasodilatation is exceeded (rCVR becomes a constant) and rCBF therefore drops in tandem with rCPP. To meet their metabolic demands, neurones must "extract more oxygen" from the passing blood - OEF increases.

Stage 3 (End-organ compromise)	If rCBF continues to fall to the extent that the brain can no longer compensate by increases in OEF, end-organ dysfunction occurs (TIA). If this situation persists, permanent end-organ damage (stroke) occurs.

The diagnosis of CMP should be considered in patients with orthostatic stroke symptoms and significant cerebrovascular stenosis. Patients with CMP have an increased risk of progression to stroke as compared to patients who have carotid occlusion without cerebral haemodynamic impairment. The prospective St. Louis Carotid Occlusion Study (STLCOS) followed 74 patients with carotid occlusion, 32 with misery perfusion, and 42 without any of the previous conditions. Over a mean follow-up period of 31.5 months, 11 patients in the study group went on to develop ipsilateral cerebral infarct compared with two in the control group (p = 0.04) [4]. This association between cerebral infarction, particularly in the watershed territories, and cerebral haemodynamic impairment has since been described in a number of cerebral perfusion imaging studies [5].

## Case presentation

**Figure 1:**
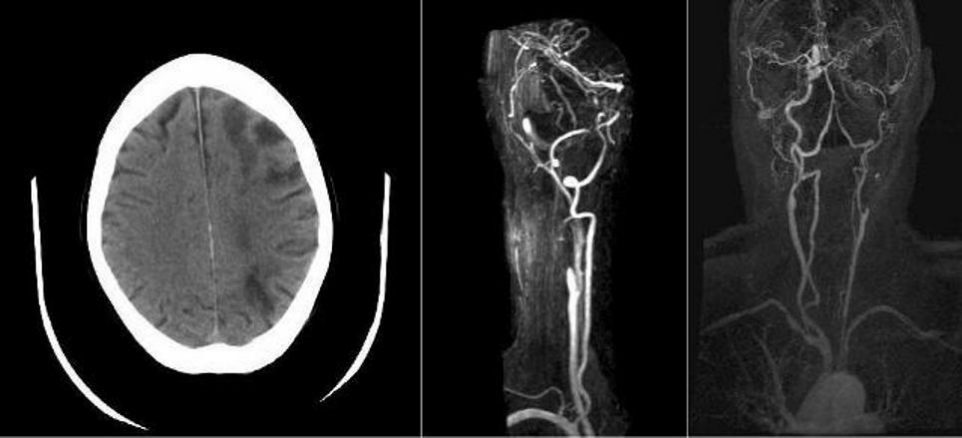
**Watershed infarcts in the anterior cortical, posterior cortical and internal watershed territories of the left cerebral hemisphere**. Magnetic resonance angiogram shows complete occlusion of the left internal carotid artery.

A 50-year-old Caucasian man was admitted with an acute right hemiparesis, affecting his arm, leg and face, and a right homonymous hemianopia. His vascular risk factors included a 25 pack-year smoking history and a previous myocardial infarction. An initial computed tomography (CT) head scan and magnetic resonance imaging (MRI) scan showed a left parieto-occipital infarction in the posterior cortical watershed territory. A carotid ultrasound Doppler scan showed a complete occlusion of his left internal carotid artery (ICA) which was confirmed on magnetic resonance angiography (Figure 1). Left carotid dissection was suspected but could not be proven as a precipitant for his arterial occlusion because no intramural haematoma was visible via a standard neck MRI. He was discharged after making a good recovery at 12 days after he was admitted. He was started on aspirin, dipyridamole and simvastatin.

Two months later he was readmitted following a primary focal seizure with secondary generalisation and mild post-ictal right-sided weakness. The results of his routine blood tests, renal function, glucose, erythrocyte sedimentation rate (ESR), Chest X-ray, echocardiogram (ECG) and 24-hour ECG were normal. A repeat MRI showed a new infarct in his right anterior cortical watershed territory. He made a reasonable recovery and was independently mobile within one week of admission.

Over the following year he suffered numerous collapses. He described these as comprising right-sided weakness and jerking movements prior to a loss of consciousness. On each occasion he demonstrated good recovery of physical function although subtle cognitive impairment was noted by serial mini-mental state examination on subsequent outpatient visits. A diagnosis of post-stroke epilepsy was made and he was commenced on oral sodium valproate. Despite this he continued to present with recurrent seizures.

A magnetic resonance scan was performed, which confirmed the presence of numerous deep watershed infarcts in addition to his two cortical infarcts. There was persistent occlusion of his left ICA on this imaging. In an attempt to understand the aetiology of our patient's recurrent strokes, we applied a hypercapnia BOLD fMRI technique to assess his cerebrovascular reserve capacity (CVR). The technique is detailed elsewhere [6]. In brief, however, serial magnetic resonance scans sensitised to tissue oxygenation (blood oxygenation level dependent [BOLD] contrast) are obtained during periods of normocapnia and hypercapnia (during inhalation of 10% CO_2_). Using these images, CVR maps are generated, where an increase in BOLD signal is used as a marker of cerebral vasodilatory capacity.

**Figure 2:**
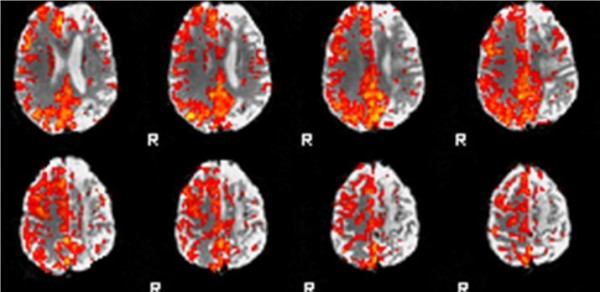
**Cerebrovascular reserve map of our patient showing left internal carotid artery occlusion and recurrent symptoms**. Note the loss of reactivity in the left hemisphere following CO2 inhalation. This is most prominent in the watershed territories.

Our patient demonstrated a significant loss of CO_2_-induced BOLD reactivity in the hemisphere distal to his carotid occlusion, suggesting maximal compensatory vasodilation at rest (Figure 2). This represents stage 2 cerebral haemodynamic impairment. He was offered an extracranial-intracranial vascular bypass surgery (EC-IC bypass) but declined it.

## Discussion

Our patient underwent a hypercapnia fMRI technique as part of ongoing research at our institution. The use of this technique has been validated in volunteers and, increasingly, in patients [6]. The result of this scan showed a marked impairment in our patient's CO_2 _reactivity in the hemisphere distal to his carotid artery occlusion. It is not possible to comment on oxygen extraction fraction from these CVR maps, but the observed change allows us to infer stage 2 cerebral haemodynamic impairment (failure of cerebrovascular autoregulation). In the context of carotid occlusion and normal BOLD increase in his contralateral hemisphere, we can further infer maximal compensatory vasodilation at rest. Whether this vasodilatation suffices to maintain cerebral perfusion via increased oxygen extraction, hence preventing progression to stage 3 cerebral haemodynamic impairment, cannot be inferred from these images.

PET scanning is unique because it provides absolute measurements for both OEF and CBF, but is cumbersome and not widely available. Other modalities, such as the BOLD-contrast fMRI used here, establish a measurement of rCBF either by comparing the affected hemisphere to the contralateral (normally perfused) hemisphere or by utilising normative reference ranges. Single Photon Emission CT (SPECT) and Xenon-enhanced CT have also been validated for clinical diagnosis of misery perfusion. Dynamic contrast enhanced CT, MRI perfusion imaging and arterial spin labelling MR techniques are newer, promising and less invasive techniques to assess rCBF [7].

There is some uncertainty surrounding the aetiology of our patient's recurrent collapses. Limb-jerking transient ischaemic attacks (TIAs) have been described in the literature and are characterised by brief, involuntary, coarse movements of the limbs. An association with carotid occlusion has been demonstrated and Han *et al*. [8] have described the failure of cerebrovascular autoregulation in these patients. However, TIAs are not associated with the loss of consciousness or with secondary generalisation and so would not fully explain this presentation.

Post-stroke seizures, by contrast, are common and are frequently characterised by generalisation and loss of consciousness. Stroke is reported to be an aetiological factor in 45% of seizures in patients over the age of 60 [9] and 5% to 20% of people who have strokes will go on to develop seizures [10]. Early post-stroke seizures, occurring up to two weeks after stroke, are postulated to result from the accumulation of intracellular calcium and sodium and extracellular glutamate during acute ischemic injury. Late seizures occurring after this time are thought to be a consequence of long-term scarring and gliosis [10]. The accumulation of new ischaemic events on repeat MRI, along with the demonstrated cerebral haemodynamic impairment, raises the possibility that our patient's seizures were a consequence of ongoing cerebral ischaemia rather than of chronic gliotic change, hence CMP is a possible explanation. It is, however, impossible to make this association with any degree of certainty.

Attempts to treat patients with CMP have focused on the augmentation of rCBF, predominantly by extracranial-intracranial vascular anastomosis (EC-IC bypass). Isolated case studies have reported the resolution of symptomatic CMP following cerebral angioplasty for intracranial arterial stenosis [11] but this procedure remains largely untested. EC-IC bypass can be used to describe a number of possible approaches but research has focused predominantly on superficial temporal artery to middle cerebral artery (STA-MCA) anastomosis. The EC/IC bypass study was a large multicenter randomized trial of STA-MCA anastomosis with primary end-points of 30-day stroke mortality and stroke incidence. A total of 1377 patients were recruited and 663 were randomized to receive EC-IC bypass. Patients randomized to the treatment group performed less well on all outcome measures despite having high graft patency rates [12]. A fundamental weakness of the study was the recruitment of patients with carotid or MCA occlusion without reference to cerebral perfusion studies, which has made it impossible to conduct subgroup analyses evaluating the efficacy of the procedure in patients with radiologically confirmed CMP.

The data from STLCOS regarding the prognosis in misery perfusion and emerging imaging techniques have provided justification for a reevaluation of EC-IC bypass. Further support has come from imaging studies demonstrating the resolution of CMP in small cohorts undergoing EC-IC bypass [13]. Two large randomized multicenter trials are underway and the publication of results is awaited [14,15]. Early intimations from the Japanese Extracranial-intracranial-bypass Trial (JET), however, have been positive, thus suggesting that data from that trial may support the intervention [15].

## Conclusion

CMP is an important differential diagnosis in post-stroke patients presenting with recurrent collapses. The hypercapnic-BOLD contrast fMRI procedure described in this case report represents a promising new imaging modality, without the resource implications of PET scanning from which cerebral misery perfusion may be inferred. Pending data from international trials may lead to an increased profile for misery perfusion in the near future if EC-IC bypass is demonstrated to be effective.

## Consent

Written informed consent was obtained from our patient for publication of this case report and any accompanying images. A copy of the written consent is available for review by the Editor-in-Chief of this journal.

## Ethical Approval

The research project discussed in this article was approved by the Derby NHS Research Ethics Committee.

## Competing interests

The authors declare that they have no competing interests.

## Authors' contributions

AG, OD and SM reviewed our patient's case notes and the current literature on cerebral misery perfusion and drafted the manuscript. SG and DA conducted the BOLD-MRI imaging, provided radiographic images, reported on these, and provided the outlines of radiological procedures incorporated in the manuscript. All authors read and approved the final manuscript.
